# Prediction of Tumor Complete Response Using Genomic and Clinical Data in Solid Cancer Undergoing Definitive Radiation Therapy

**DOI:** 10.3390/ijms27208991

**Published:** 2026-10-10

**Authors:** Jun Young Chang, Seung-Hyun Jung, Dong Soo Lee

**Affiliations:** 1 Department of Medical Sciences, Graduate School, The Catholic University of Korea, Seoul 06591, Republic of Korea; 2 Catholic Research Institute for Human Genome Polymorphism, College of Medicine, The Catholic University of Korea, Seoul 06591, Republic of Korea; 3 Department of Biochemistry, College of Medicine, The Catholic University of Korea, Seoul 06591, Republic of Korea; 4 Department of Radiation Oncology, College of Medicine, The Catholic University of Korea, Seoul 06591, Republic of Korea

**Keywords:** next-generation sequencing, esophageal cancer, head and neck cancer, radiation therapy, radiogenomics, response, rectal cancer

## Abstract

In this retrospective, single-center observational study, targeted next-generation sequencing (NGS) was performed on pre-radiation therapy (RT) tumor specimens to identify genomic features associated with RT response and clinical outcomes. This study included 77 eligible patients with esophageal squamous cell carcinoma (ESCC, *n* = 23), non-human papilloma virus-related head and neck squamous cell carcinoma (HNSCC, *n* = 24), and rectal adenocarcinoma (RAC, *n* = 30) treated between 2014 and 2020. Treatment was employed for definitive aim (chemoradiotherapy or pre-operative chemoradiotherapy followed by radical surgery). Treatment response was assessed using clinical and pathological criteria and was dichotomized as complete response (CR) versus non-CR. Targeted NGS was performed using a customized panel composing 161 genes. A total of 1263 nonsynonymous somatic variants were identified across 153 genes. The most commonly altered genes were *TP53* (70.1%), *APC* (41.6%), *NOTCH1* (41.6%), *KMT2C* (39.0%), *NOTCH3* (36.4%), and *NOTCH2* (35.1%). Overall, 25 (32.5%) and 52 patients (67.5%) were classified as CR and non-CR, respectively. *APC* (*P* = 0.008), *KMT2C* (*P* = 0.018), and *KRAS* (*P* = 0.012) alterations were associated with non-CR, whereas *CD274* (*P* = 0.003), *MSH3* (*P* = 0.011), and *NOTCH3* (*P* = 0.048) alterations were associated with CR. *KRAS* alterations occurred exclusively in RAC, and most concurrent *APC*–*KMT2C* alterations in non-CR tumors were also observed in RAC, suggesting that these associations may partly reflect tumor type-specific genomic backgrounds. An exploratory pooled model incorporating age and six candidate genomic variables achieved a cross-validated area under the curve of 0.867 for CR prediction. In survival analysis, alterations in *CCND1*, *CXCL9*, *ERCC6*, *LIF*, and *RAD51B* were independently associated with poor recurrence-free survival. Pre-RT tumor genomic profiling identified candidate features associated with RT response and recurrence-free survival in this heterogeneous cohort. The pooled genomic associations and prediction model should not be interpreted as pan-cancer biomarkers or predictors and require validation in larger, independent, cancer-specific cohort.

## 1. Introduction

Radiation therapy (RT) is a central modality in modern cancer care. It has been estimated that approximately half of all patients with cancer will require RT at least once during their disease course and that RT contributes to around 40% of cancer cures [1,2]. Despite continuous advances in cancer treatment and the widespread adoption of standardized treatment protocols, clinical responses to RT remain heterogeneous [3,4]. While some patients achieve complete tumor remission, others experience locoregional recurrence or distant metastasis due to intrinsic or acquired radioresistance, ultimately leading to poorer survival outcomes [4]. This persistent inter-patient variability highlights an unmet need for biomarkers that can stratify patients according to radiosensitivity and support precision RT.

With the rapid evolution of high-throughput sequencing technologies and bioinformatic tools, radiogenomics has emerged as an approach to link genomic variation to RT response and outcomes [3]. At the cellular level, ionizing radiation induces a spectrum of DNA lesions, among which DNA double-strand breaks are major cytotoxic events. The DNA damage response (DDR) network orchestrates damage sensing, cell-cycle control, and repair, thereby strongly influencing radiosensitivity and radioresistance [4,5,6]. Consistent with this, alterations in DDR genes and pathways, including *TP53*, *ATM*, and *BRCA1/2*, have been implicated in treatment failure across multiple tumor types [7]. In addition to direct DNA damage, oxidative stress, inflammation, oncogenic signaling, and tumor-immune interactions are also important determinants of radiation response [8,9]. In particular, genomic dysregulation of NRF2-KEAP1 signaling has been associated with clinical radioresistance, including higher local recurrence after RT in *KEAP1/NFE2L2*-mutant tumors [10]. Nevertheless, previous studies evaluating associations between tumor genomic features and RT response have varied in study design, tumor types, sequencing strategy, and gene content, contributing to heterogeneous findings and limiting clinical translation [2,11,12,13,14].

Targeted next-generation sequencing (NGS) offers a practical strategy for biomarker discovery and potential clinical implementation by enabling high-depth coverage of genes of interest. In this study, we developed a customized targeted NGS panel enriched for DDR pathways, along with cancer-associated and immune-related pathways, to interrogate pre-RT tumor specimens from patients treated with definitive or preoperative RT across multiple solid tumor types. Specifically, we aimed (1) to identify genomic alterations associated with complete response (CR) versus non-CR after RT, (2) to characterize pathway-level differences linked to RT response, and (3) to develop an exploratory predictive model for CR using somatic alterations detected by targeted sequencing. We further explored the prognostic relevance of clinical and genomic factors for recurrence-free survival (RFS) and overall survival (OS). Given the distinct biological backgrounds of the included tumor types, the pooled analyses were intended to explore candidate genomic features and pathways associated with RT response across tumor types rather than to establish tumor-specific biomarkers. These analyses provide useful information on genomic features associated with RT response across tumor types.

## 2. Results

### 2.1. Patient Characteristics

A total of 77 patients who underwent RT were included in this study. The clinicopathological and treatment characteristics, including detailed clinical T, *n* and M categories and baseline M status, are summarized in Table 1. The cohort consisted of 61 males (79.2%) and 16 females (20.8%). Following treatment, 25 patients (32.5%) achieved CR, whereas 52 patients (67.5%) were categorized into the non-CR group. The median age of the entire cohort was 68 years, with the CR group (median 69, range 57–85) being significantly older than the non-CR group (median 65.5, range 44–81; *P* = 0.015). Regarding primary tumor sites, the final cohort included ESCC (*n* = 23, 29.9%), HNSCC (*n* = 24, 31.2%), and RAC (*n* = 30, 39%). The distributions of clinical T and *N* categories, gender, histological type, biologically effective dose (BED), and concomitant chemotherapy did not differ significantly between the CR and non-CR groups. However, the non-CR group exhibited a significantly higher recurrence rate (29/52, 55.8%) compared to the CR group (7/25, 28.0%; *P* = 0.022), supporting the clinical relevance of treatment response.

### 2.2. Genomic Landscape and Response-Associated Alterations

**Figure 1:**
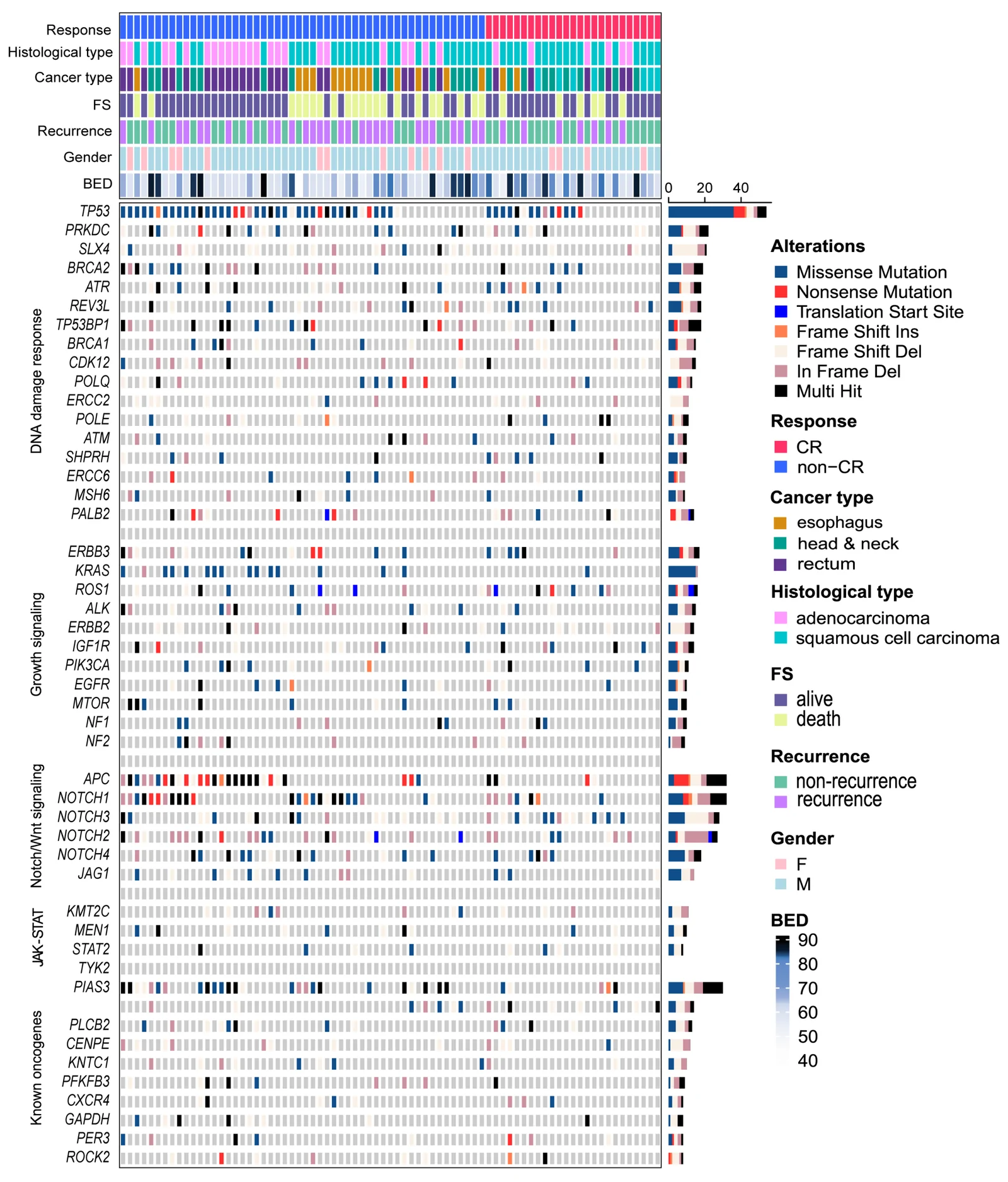
Heatmap showing genomic alterations in genes cataloged in the Catalogue of Somatic Mutations in Cancer (COSMIC) database. FS, final status; BED, biological effect dose; CR, complete response.

To profile the genomic landscape, targeted deep sequencing was performed on pre-RT tumor specimens. The sequencing achieved a high mean coverage depth of 3095.4× (range 1780.5–7255.4×). After stringent filtering, a total of 1263 nonsynonymous mutations were identified across 153 genes (Table S1). Among these, recurrent genetic alterations were identified in 47 genes with a mutation frequency ≥ 10%, including 27 established cancer-related genes cataloged in the COSMIC database (Figure 1). The most frequently mutated gene in the overall cohort was *TP53* (*n* = 54, 70.1%), followed by *APC* (*n* = 32, 41.6%), *NOTCH1* (*n* = 32, 41.6%), *KMT2C* (*n* = 30, 39.0%), *NOTCH3* (*n* = 28, 36.4%), and *NOTCH2* (*n* = 27, 35.1%). To present cancer type-specific genomic patterns separately, mutation profiles were visualized for ESCC, HNSCC, and RAC (Figure S1). The distribution and frequency of recurrent alterations differed among the three tumor types.

Quantitatively, there was no significant difference in the total number of somatic mutations between the CR and non-CR groups (*P* = 0.130). However, distinct differences were observed at the individual gene level. Based on univariate analysis, six genes (*APC*, *CD274*, *KMT2C*, *KRAS*, *MSH3*, and *NOTCH3*) were significantly associated with RT response (Table 2). Specifically, mutations in *APC* (*P* = 0.008, OR = 4.32, 95% confidence interval [CI] 1.41–13.25), *KRAS* (*P* = 0.012, OR = 9.73, 95% CI 1.21–78.54), and *KMT2C* (*P* = 0.018, OR = 3.70, 95% CI 1.21–11.36) were significantly enriched in the non-CR group, suggesting a potential role in radioresistance. In contrast, mutations in *CD274* (*P* = 0.003, OR = 0.05, 95% CI 0.002–0.88), *MSH3* (*P* = 0.011, OR = 0.06, 95% CI 0.003–1.23), and *NOTCH3* (*P* = 0.048, OR = 0.37, 95% CI 0.14–1.01) were more frequently observed in the CR group. Pairwise analysis showed that concurrent *APC*–*KMT2C*, *APC*–*KRAS*, and *KMT2C*–*KRAS* alterations were more frequently observed in the non-CR group than in the CR group (16/52 [30.8%] vs. 1/25 [4.0%], 13/52 [25.0%] vs. 1/25 [4.0%], and 8/52 [15.4%] vs. 0/25 [0%], respectively; Table S2). The corresponding unadjusted Fisher’s exact *P*-values were 0.008, 0.029, and 0.048. All *KRAS* alterations occurred in RAC, and 12 of the 16 concurrent *APC*–*KMT2C* alterations in the non-CR group were also observed in RAC. *CD274* and *MSH3* alterations were observed only in the CR group but were infrequent, limiting further assessment of overlapping patterns.

At the variant level, the non-CR group was characterized by enrichment of loss-of-function (LoF) mutations in tumor suppressors and epigenetic regulatory genes (Table S1). In APC, most variants in non-CR tumors were truncating mutations, including nonsense and frameshift variants such as p.R1450X and p.S1346X, suggesting that *APC* inactivation may be associated with reduced RT sensitivity. Similarly, *KMT2C* exhibited a diverse spectrum of truncating mutations that were exclusively or predominantly observed in the non-CR group. *KRAS* mutations in the non-CR group were concentrated at oncogenic hotspots (p.G12D and p.G13D), predominantly in patients with RAC, supporting a possible contribution of RAS-MAPK signaling to RT resistance. In contrast, the CR group harbored distinct mutational profiles involving genes related to immune regulation and DNA repair, with all identified mutations in *CD274* (PD-L1) and *MSH3* found exclusively in this group (Figure S2A). For *NOTCH3*, variant-level mapping identified 32 variants in 28 tumors, including 14 frameshift deletions, one nonsense variant, 12 missense variants, and five in-frame deletions (Figure S1A and Table S1). At least one potentially truncating *NOTCH3* variant was identified in 5 of 25 CR tumors (20.0%) and 10 of 52 non-CR tumors (19.2%), with no significant difference between the groups.

### 2.3. Functional Enrichment and Pathway Analysis

To investigate biological processes potentially underlying differential RT response, we performed pathway enrichment analysis (Figure S2B). Several pathways, including ‘response to radiation’, ‘regulation of catalytic activity’, and ‘regulation of MAPK cascade’, were significantly altered regardless of RT responsiveness. However, the CR and non-CR groups exhibited distinct enrichment patterns. The CR group showed a significant enrichment in pathways related to active cell turnover, such as ‘cell population proliferation’ and ‘growth’ (Table S3), suggesting that tumors with higher baseline proliferative activity may be associated with increased susceptibility to the DNA-damaging effects of ionizing radiation. In contrast, the non-CR group was characterized by an enrichment of pathways associated with therapeutic evasion and survival. Notably, ‘pathways in cancer’, and ‘EGFR tyrosine kinase inhibitor resistance’ were the most significantly overrepresented terms in non-CR tumors (Table S4). In addition, enrichment of ‘DNA repair’ and ‘platinum drug resistance’ pathways in the non-CR group provides a genomic context for reduced RT responsiveness. Collectively, these findings suggest that enhanced DNA damage recovery and activation of alternative survival-related signaling pathways may contribute to treatment failure. Pathway enrichment analyses were also performed separately for ESCC, HNSCC, and RAC (Figure S3 and Table S5). ‘Pathways in cancer’ and ‘regulation of MAPK cascade’ were among the enriched terms in all three tumor types, whereas the remaining enriched terms differed by tumor type.

### 2.4. Prognostic Factors

To identify prognostic factors, we performed univariate Cox regression analysis (Table 3). Two clinical variables, concomitant chemotherapy and treatment response, were significantly associated with RFS. In addition, ten genomic alterations (*CCND1, CTNNB1, CXCL9*, *ERCC6*, *FANCD2*, *LIF*, *MIEN1*, *PDCD1*, *RAD51B*, and *SLC2A3*) were significantly associated with poor RFS. Kaplan–Meier analysis showed significant differences in RFS for all clinical and genomic variables identified in the univariate Cox regression analysis (Figure S4). In the multivariate analysis including all significant candidates from the univariate analysis, alterations in *CCND1* (hazard ratio [HR] = 5.44, 95% CI 1.60–18.42, *P* = 0.007), *CXCL9* (HR = 579.59, 95% CI 35.41–9487.41, *P* < 0.001), *ERCC6* (HR = 5.07, 95% CI 1.92–13.35, *P* = 0.001), *LIF* (HR = 4.80, 95% CI 1.77–13.0, *P* = 0.002), and *RAD51B* (HR = 6.81, 95% CI 2.14–21.62, *P* = 0.001) remained independent prognostic factors for poor RFS (Table 3). Conversely, concomitant chemotherapy (HR = 0.12, 95% CI 0.04–0.36, *P* < 0.001) was confirmed as an independent favorable prognostic factor for RFS.

### 2.5. Development of an Integrated Clinical and Genomic Model for Predicting RT Response

To develop the RT response prediction models, candidate predictors were selected from the univariable comparisons of the CR and non-CR groups presented in Table 1 and Table 2. Age, dichotomized at 68 years, was selected as the pre-treatment clinical predictor. Although recurrence met the selection criterion, it was excluded because it occurred after the initial response assessment. Six response-associated genomic variables (*APC*, *CD274*, *KMT2C*, *KRAS*, *MSH3*, and *NOTCH3*) were selected as genomic predictors. The integrated model included age and all six genomic variables simultaneously in a multivariable logistic regression model. The predictive performance of three models (clinical variable only, genomic variable only, and an integrated model) was evaluated using a 10-fold CV approach.

**Figure 2:**
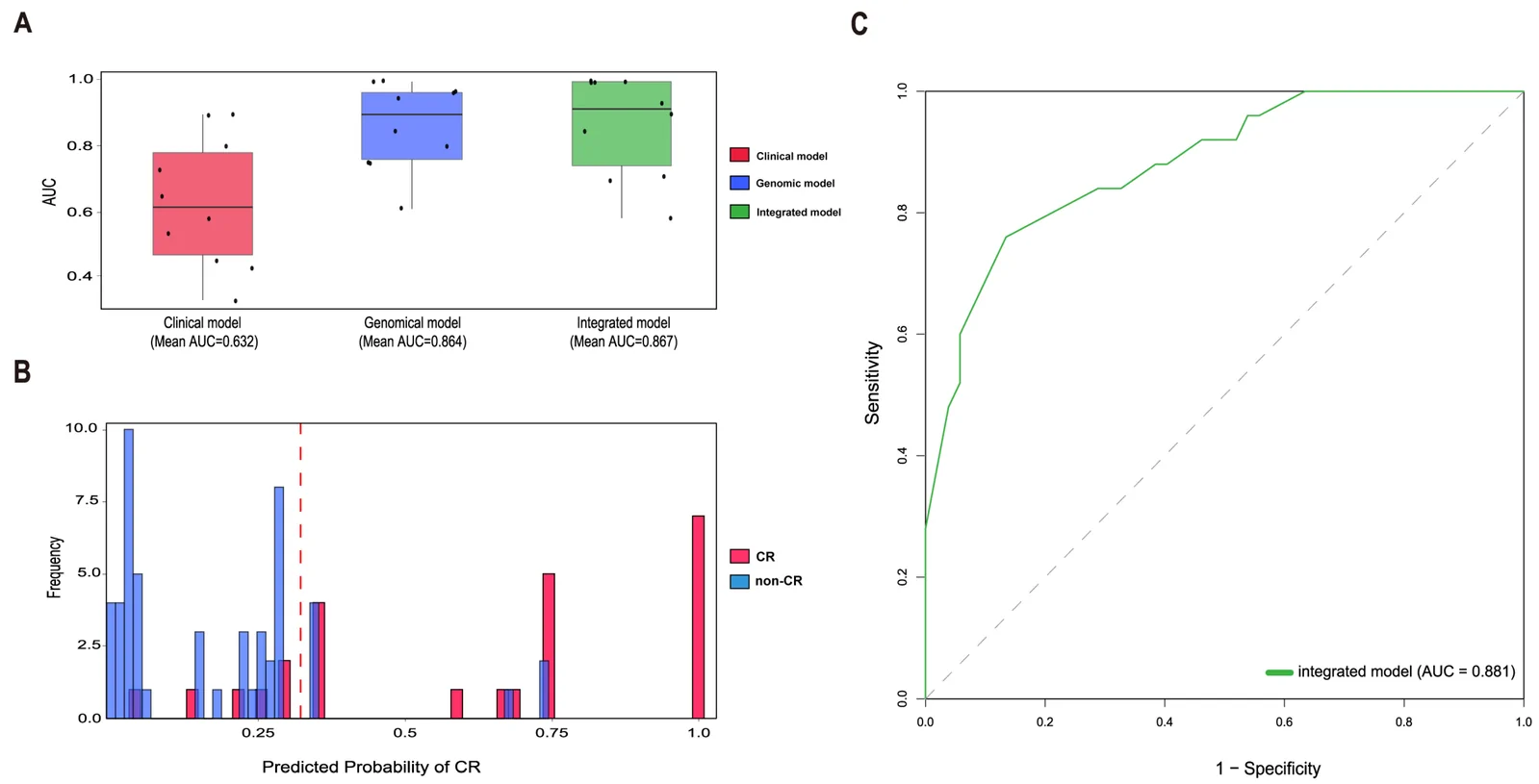
(**A**) Bar plots showing cross-validation AUC-ROC comparing clinical (red), genomic (blue), integrated logistic regression models (green). (**B**) Prediction probability distribution for CR (red) and non-CR (blue) groups. (**C**) AUC-ROC curve of integrated logistic regression.

The integrated model demonstrated the best overall performance compared to the individual models, achieving the highest CV-adjusted AUC of 0.867, a sensitivity of 94.0%, and an accuracy of 82.9%. The clinical model and genomic model yielded lower CV-AUCs of 0.632 and 0.864, respectively (Figure 2A). These suggest that integrating clinical and genomic variables may provide a more comprehensive framework for RT response prediction, although the incremental gain over the genomic model alone was limited. The final integrated prediction formula was derived from the entire cohort (*n* = 77) to determine the definitive regression coefficients. Categorical genomic variables were coded as 1 for alteration and 0 for wild-type, and age was coded as 1 for ≥68 years and 0 for <68 years. The logit (z) and the predicted probability (P) of achieving CR were defined as follows: z = −0.862 + (0.281 × Age ≥ 68 status) − (0.381 × *APC* status) + (20.241 × *CD274* status) − (2.123 × *KMT2C* status) − (2.147 × K*RAS* status) + (19.820 × *MSH3* status) + (1.641 × *NOTCH3* status)

P(CR) = 1/(1 + exp(−z))(1)

The optimal probability threshold for classifying patients into the CR group was 0.328 based on the Youden index (Table S6). Using this threshold, the predicted probabilities showed a clear separation between the CR and non-CR groups (Figure 2B), with an AUC of 0.881, a sensitivity of 76.0%, a specificity of 86.5%, and an overall accuracy of 83.1% (Figure 2C). As a subgroup analysis, the pooled model was applied separately to ESCC, HNSCC, and RAC. The AUCs were 0.964, 0.829, and 0.906, respectively (Table S7). Because these estimates were obtained from subgroups of the model development cohort, they do not constitute external validation and should be interpreted cautiously.

## 3. Discussion

The clinical implementation of personalized RT remains a significant challenge due to the complex interplay between tumor genetics and radiation response. In this study, we identified distinct pre-treatment genomic features associated with RT responsiveness and developed an integrated clinical-genomic model for CR prediction. Although the overall mutational burden did not differ significantly between the CR and non-CR groups, gene-level analyses identified differences in the frequencies of *APC*, *KRAS*, *KMT2C*, *CD274*, *MSH3*, and *NOTCH3* alterations between the two groups. These results suggest that pre-treatment tumor genomic features related to DDR, oncogenic signaling, and tumor-immune interactions may be associated with RT response in this cohort [4]. ESCC, HNSCC, and RAC have distinct genomic backgrounds, and the cancer type stratified analyses showed differences in recurrent alterations and enriched pathways. Therefore, some pooled gene-level associations may be influenced by tumor type composition. The identified alterations should be interpreted as candidate features associated with RT response in this cohort rather than as tumor-independent biomarkers.

Mutations in *APC*, *KRAS*, and *KMT2C* were more common in the non-CR group, suggesting a possible association with reduced RT response. In our cohort, *KRAS* mutations were concentrated at canonical hotspots (p.G12D/G13D), mainly in RAC, suggesting that constitutive RAS-MAPK signaling may have contributed to resistance to RT. Consistent with this, favorable chemoradiosensitivity signatures in rectal cancer have been associated with a lower frequency of *KRAS* mutation [12]. Previous studies have also reported that oncogenic *KRAS* can promote radioresistance through stem-like programs, osteopontin-EGFR signaling, and enhanced DNA double-strand break repair through NRF2-53BP1-mediated non-homologous end joining [15,16]. Although direct evidence between *KMT2C* alteration and RT responsiveness remains limited, a stage III non-small cell lung cancer (NSCLC) chemoradiation study identified *KMT2C*/*KMT2D* alterations as negative predictors of local-regional control and survival [17]. This supports the possibility that chromatin-regulatory defects involving *KMT2C* may contribute to treatment resistance in selected settings. The enrichment of *APC* alterations in the non-CR group is also notable but should be interpreted with caution because the current evidence does not support *APC* as an established pan-cancer determinant of radioresistance. A more plausible explanation is that *APC* alterations may reflect Wnt/β-catenin pathway dysregulation, which has been associated with poor response to neoadjuvant chemoradiotherapy in rectal cancer [18,19]. In our cohort, *APC* may therefore mark a lineage-related Wnt-driven resistance phenotype rather than a universal RT-resistance mechanism.

The CR-associated alterations also provide important clues. The association of *MSH3* with CR suggests that DNA repair-related vulnerability, rather than mutational burden alone, may influence RT response. This is important because mismatch-repair deficiency does not show a uniform radiation phenotype across models or dose conditions [20]. Similarly, a previous study reported possible radioresistance in some MMR-deficient settings, but the evidence remains insufficient for clinical conclusions [21]. *CD274* points to the role of tumor-immune interaction in RT responsiveness. In our cohort, all identified *CD274* mutations were observed in CR tumors. This pattern is consistent with immune-active response states described in rectal cancer, including enrichment of CXCL9/CXCL10/STAT1-related programs, higher Th1/Tex/naïve CD4-associated immune states, and reduced immunosuppressive macrophage features [12,22]. A single-cell breast cancer study also showed that radiosensitivity and radioresistance are accompanied by distinct immune checkpoint crosstalk patterns [23]. Together, our results support the view that both DNA repair context and tumor-immune state contribute to CR after RT.

Regarding *NOTCH3*, our result is directionally consistent with a previous targeted sequencing study showing that Notch pathway mutation was associated with radiosensitivity at the pathway level in a heterogeneous solid tumor cohort [13]. On the other hand, multiple experimental studies have shown that radiation-induced Notch activation promotes radioresistance and that Notch inhibition enhances radiosensitivity [24,25]. This discrepancy may have several explanations. First, pathway-level alteration and a single-gene event are not equivalent. Second, a *NOTCH3* mutation does not necessarily indicate pathway activation, and its functional consequence may differ according to whether the mutation is activating, truncating or neutral. In our cohort, 15 *NOTCH3* variants were frameshift or nonsense mutations compatible with possible LoF. Potentially truncating variants occurred at similar frequencies in the CR and non-CR groups, providing no evidence for a LoF-specific association with CR. The functional effects of the remaining variants are uncertain. Third, our cohort included multiple tumor types, and the effect of *NOTCH3* alterations may depend on tissue context and co-mutations. Therefore, our data supports the relevance of Notch biology but does not support a uniform interpretation of *NOTCH3* alterations across cancers.

The concurrent alteration patterns should be interpreted in the context of tumor type. The *APC*–*KRAS* and *KMT2C*–*KRAS* combinations occurred exclusively in RAC, and most concurrent *APC*–*KMT2C* alterations in non-CR tumors were also observed in RAC. These patterns may therefore reflect the genomic background of RAC rather than a common functional interaction among the three genes. Previous studies have linked *KRAS* alterations to radioresistance [15,16], *KMT2C*/*KMT2D* alterations to adverse outcomes after chemoradiation [17], and APC/Wnt–β-catenin pathway dysregulation to reduced response in rectal cancer [18,19]. However, the present data do not establish functional interactions or an RT-specific synergistic effect among these alterations. The small numbers of *CD274* and *MSH3* alterations also limited assessment of their biological relationships with the other response-associated genes.

An important practical implication of our study is the feasibility of an integrated prediction model. The final model combined age with six genomic variables and showed excellent discriminative performance. Although the incremental improvement over the genomic-only model was modest, the integrated model still provides a clinically interpretable framework using pre-treatment information. This is important because radiogenomic biomarkers are unlikely to be clinically useful if restricted to isolated single-gene associations. Their value will more likely come from multivariable models that combine molecular and clinical information [4]. Previous studies in heterogenous solid tumors and stage III NSCLC have also suggested that clinico-genomic integration can improve RT-response stratification [14,17].

This study has some limitations. First, it was a retrospective, single-center study with a small cohort comprising three biologically distinct tumor types. Although cancer type stratified analyses were performed, the limited subgroup sizes precluded adequately powered tumor-specific association analyses and prediction model development, and the pooled results may partly reflect tumor type composition. Second, matched normal tissues were unavailable, and variant calling was therefore performed in tumor-only mode. Although stringent post-filtering was applied, uncertainty regarding somatic status cannot be completely excluded. Third, the prediction model was internally cross-validated but not externally validated. Validation in larger, independent, cancer-specific cohorts with comparable pre-treatment genomic and RT-response data is required. Fourth, no functional experiments were performed to determine whether the identified alterations directly affect radiosensitivity or radioresistance. Finally, targeted DNA sequencing may not fully capture transcriptomic, epigenetic, or microenvironmental determinants of radiation response.

In conclusion, our study identified candidate genomic features associated with RT response in a heterogeneous cohort comprising ESCC, HNSCC, and RAC. Because several key associations were concentrated in RAC, the pooled clinical-genomic model should be interpreted as specific to this cohort rather than as a pan-cancer predictor. Validation in larger, independent, cancer-specific cohorts is required before clinical application.

## 4. Materials and Methods

### 4.1. Study Population and Eligibility

Institutional review board approval (IRB No: UC19EESE0127) was obtained before study initiation. The inclusion criteria were as follows: (1) patients who provided written informed consent and had an approved human-derived material research form; (2) patients with adequate pre-RT tumor samples; and (3) patients who met the pathological eligibility criteria upon examination. The exclusion criteria were: (1) patients with unavailable information for treatment response evaluation; (2) patients who were lost to follow-up; and (3) patients who did not complete the planned radiation therapy (RT) course due to treatment interruption. 

Four cancer types were initially considered in this study: esophageal squamous cell carcinoma (ESCC), non-human papilloma virus-related head and neck squamous cell carcinoma (HNSCC), rectal adenocarcinoma (RAC), and stomach cancer (adenocarcinoma, signet ring cell carcinoma). Between 2014 and 2020, a total of 91 patients were initially eligible for inclusion. Among these, three patients with esophageal cancer and 11 patients with stomach cancer were excluded because the treatment intent was palliative. Consequently, a total of 77 patients treated with curative intent were included in the final analysis.

### 4.2. Patient Characteristics and Treatment

All patients were treated with curative intent, and standard treatment protocols, including RT doses and combination chemotherapy, were applied to all patients. For ESCC and HNSCC, definitive chemoradiation therapy (CRT) or RT was administered according to tumor stage and established treatment guidelines. For RAC, preoperative CRT followed by curative total mesorectal excision surgery was performed according to the standard treatment guidelines for locally advanced stages. In ESCC, patients underwent either definitive CRT/RT or preoperative CRT followed by curative surgery, as determined by multidisciplinary tumor board decisions.

### 4.3. Response Evaluation and Follow-Up

Treatment response was evaluated based on pre-treatment and post-treatment imaging studies. Imaging studies included standard computed tomography (CT) and positron emission tomography CT (PET–CT), which were used for initial staging and response assessment at 3 months after treatment. For patients who underwent definitive CRT/RT, treatment response was assessed according to the Response Evaluation Criteria in Solid Tumors (RECIST) version 1.1. For patients who underwent preoperative CRT/RT, response evaluation was based on pathological staging of the resected surgical specimens. Complete response (CR) was defined as complete tumor regression or pathological findings of ypT0 or ypTis with no residual metastatic lymph nodes (ypN0). Partial response (PR) was defined as downstaging of T and N stages on pathological examination compared with the clinical pre-RT stage. Stable disease (SD) was defined as no change in pathological stage relative to the clinical pre-RT stage, whereas progressive disease (PD) was defined as upstaging of T and N stages on pathological examination compared with the clinical pre-RT stage. To harmonize response assessment across definitive and preoperative treatment settings, response was ultimately categorized as CR or non-CR for the main analyses. Patients were routinely followed up at 1 month after completion of treatment and every 1–3 months thereafter. At each follow-up visit, medical history review, physical examination, and imaging studies were performed.

### 4.4. Targeted Next-Generation Sequencing and Identification of Genomic Variants

Eligible pre-RT tumor samples were retrieved from the institutional pathology archives. All specimens were preserved in formalin-fixed, paraffin-embedded (FFPE) blocks and reviewed by an experienced pathologist to ensure histological suitability. Genomic DNA was isolated from 10-μm-thick FFPE tissue sections using the RecoverAll™ Total Nucleic Acid Isolation Kit (Thermo Fisher Scientific, Waltham, MA, USA) in accordance with the manufacturer’s protocol. DNA quality and quantity were assessed using an ND-1000 spectrophotometer (Thermo Fisher Scientific) and a Qubit 2.0 Fluorometer (Thermo Fisher Scientific).

Targeted sequencing and variant analysis were performed using a previously described methodological framework [26]. For the present study, a customized panel comprising 161 genes was selected to cover DNA damage response and repair pathways relevant to RT response, including mismatch repair, nucleotide excision repair, homologous recombination repair, nonhomologous end joining, and the Fanconi anemia pathway. The panel also included immune-related, cytokine-related, and established cancer-associated genes to assess genomic features beyond DNA repair that may be associated with treatment response. The complete gene list is provided in Table S8. Paired-end sequence reads were generated on the Illumina NovaSeq 6000 platform (Illumina, Inc., San Diego, CA, USA). Sequencing reads were aligned to the human reference genome (GRCh38) using the Burrows–Wheeler Aligner (version 0.7.17-r1188), and alignment quality was evaluated using Qualimap (version 2.3) [27].

Single-nucleotide variants (SNVs) and indels were identified using MuTect2 [28]. Given the absence of matched normal controls, Mutect2 (version 4.3.0) was executed in tumor-only mode using an in-house panel of normals for noise reduction. The ANNOVAR package (version 2020Jun08) was used to annotate variants located in exonic regions and to predict their functional consequences [29]. To ensure high-confidence variant calling, variants meeting the following criteria were filtered out: (1) total altered read count < 10, altered read count on either strand < 5, or strand bias > 90%; and (2) variants reported as polymorphisms in gnomAD or the Exome Aggregation Consortium with a minor allele frequency ≥ 0.1% in East Asian populations. The remaining variants were considered candidate somatic variants. For the analysis of mutation profiles in the CR and non-CR groups, only nonsynonymous variants were included. Kyoto Encyclopedia of Genes and Genomes (KEGG) pathway and Gene Ontology (GO) analyses were performed using Metascape (version 3.5), focusing on genes that are listed in the COSMIC database and mutated at a frequency of ≥10% within each group [30]. Mutation profiles and pathway enrichment results were additionally summarized according to cancer type.

### 4.5. Development of the RT Response Prediction Model

Candidate clinical and genomic predictors were screened based on the univariable comparisons of the CR and non-CR groups presented in Table 1 and Table 2. Variables with a *P*-value ≤0.05 were considered candidate predictors. Given the limited cohort size, this screening step was used to restrict the number of candidate predictors. Recurrence was excluded because it occurred after the initial response assessment. The selected pre-treatment variables were entered simultaneously and retained in the respective logistic regression models. The integrated model was constructed using multivariable logistic regression, and model performance was assessed using 10-fold cross-validation (CV). Model performance was evaluated using receiver operating characteristic (ROC) curves with the pROC package in R. Additional performance metrics, including the area under the curve (AUC), sensitivity, specificity, accuracy, and F1 score, were also calculated. Because of the limited number of patients within each subgroup, separate tumor-specific prediction models were not constructed. The pooled model was applied to each cancer type as a subgroup analysis.

### 4.6. Statistical Analysis

Continuous variables were analyzed using Student’s *t*-test or the Mann–Whitney U test according to the normality of the data distribution. Categorical variables were compared between the CR and non-CR groups using the chi-square test or Fisher’s exact test, as appropriate. Pairwise concurrent alterations among the six response-associated genes were compared between the CR and non-CR groups using Fisher’s exact test. We also applied Haldane–Anscombe correction to estimate odds ratio (OR) in the presence of zero-count cells, whereby 0.5 was added to all cells. For survival analysis, univariate and multivariate Cox regression analyses were performed to identify independent prognostic factors for recurrence-free survival (RFS) and overall survival (OS). RFS was defined as the interval from the initiation of RT to disease recurrence or the last follow-up, whereas OS was measured from RT initiation to death from any cause. Survival curves were estimated using the Kaplan–Meier method for variables that were significant in the univariate Cox regression analysis and compared using the log-rank test. All statistical tests were two-sided, and a *P*-value ≤ 0.05 was considered statistically significant. All statistical analyses were executed using R software (version 4.4.2) and IBM SPSS Statistics (version 25; IBM Corp., Armonk, NY, USA).

## Figures and Tables

**Table 1:** Clinicopathological and treatment characteristics of the study cohort.

Variable	CR (*n* = 25)	Non-CR (*n* = 52)	*P*
Age (median, range)	69 (57–85)	65.5 (44–81)	0.015
Gender, *n* (%)			0.474
Male	21 (84.0%)	40 (76.9%)	
Female	4 (16.0%)	12 (23.1%)	
Recurrence, *n* (%)			0.022
Yes	7 (28.0%)	29 (55.8%)	
No	18 (72.0%)	23 (44.2%)	
Clinical T category, *n* (%)			0.418
cT1	2 (8.0%)	1 (1.9%)	
cT2	4 (16.0%)	5 (9.6%)	
cT3	11 (44.0%)	24 (46.2%)	
cT4	8 (32.0%)	22 (42.3%)	
Clinical N category, *n* (%)			0.465
cN0	5 (20.0%)	8 (15.4%)	
cN1	11 (44.0%)	24 (46.2%)	
cN2	9 (36.0%)	15 (28.8%)	
cN3	0 (0.0%)	5 (9.6%)	
Histology, *n* (%)			0.062
Squamous cell carcinoma	19 (76.0%)	28 (53.8%)	
Adenocarcinoma	6 (24.0%)	24 (46.2%)	
BED, *n* (%)			0.333
≤63.7 Gy	11 (44.0%)	29 (55.8%)	
>63.7 Gy	14 (56.0%)	23 (44.2%)	
Concomitant chemotherapy, *n* (%)			0.266
No chemotherapy	1 (4.0%)	4 (7.7%)	
LF	3 (12.0%)	15 (28.8%)	
Capecitabine	3 (12.0%)	9 (17.3%)	
FP	8 (32.0%)	13 (25.0%)	
CDDP	10 (40.0%)	11 (21.2%)	

CR, complete response; BED, biological effective dose; Gy, gray; LF, leucovorin/5-fluorouracil; FP, 5-fluorouracil/cisplatin; CDDP; cisplatin. *P*-values were calculated using the Mann–Whitney U test for age and the chi-square test or Fisher’s exact test for categorical variables, as appropriate.

**Table 2:** Genomic alterations associated with radiotherapy response.

Gene	Alterations in CR (*n* = 25)		Alterations in Non-CR (*n* = 52)		*P*	OR (95% CI)
	Present	Absent	Present	Absent		
*CD274*	4 (16.0%)	21 (84.0%)	0(0.0%)	52 (100.0%)	0.003	0.05 (0.002–0.88)
*APC*	5 (20.0%)	20 (80.0%)	27 (52.0%)	25 (48.0%)	0.008	4.32 (1.41–13.25)
*MSH3*	3 (12.0%)	22 (88.0%)	0(0.0%)	52 (100.0%)	0.011	0.06 (0.003–1.23)
*KRAS*	1 (4.0%)	24 (96.0%)	15 (29.0%)	37 (71.0%)	0.012	9.73 (1.21–78.54)
*KMT2C*	5 (20.0%)	20 (80.0%)	25 (48.0%)	27 (52.0%)	0.018	3.70 (1.21–11.36)
*NOTCH3*	13 (52.0%)	12 (48.0%)	15 (29.0%)	37 (71.0%)	0.048	0.37 (0.14–1.01)

CR, complete response; OR, odds ratio; CI, confidence interval. *P*-value was calculated by chi-square test.

**Table 3:** Prognostic factors associated with recurrence-free survival.

Variable	Univariate COX Regression		Multivariate COX Regression	
	*P*	HR (95% CI)	*P*	HR (95% CI)
**Clinical Factors**				
non-CR (vs. CR)	0.013	2.86 (1.25–6.55)	0.152	1.87 (0.80–4.39)
Concomitant chemotherapy (Yes vs. No)	0.042	0.33 (0.12–0.96)	<0.001	0.12 (0.04–0.36)
**Genomic Factors (Altered vs. WT)**				
*CCND1*	0.011	4.70 (1.42–15.51)	0.007	5.44 (1.60–18.42)
*CTNNB1*	0.006	3.96 (1.50–10.50)	0.165	2.03 (0.75–5.49)
*CXCL9*	0.002	74.5 (4.66–1191)	8.16 × 10^−6^	579.59 (35.41–9487.41)
*ERCC6*	0.016	3.24 (1.24–8.47)	0.001	5.07 (1.92–13.35)
*FANCD2*	<0.001	11.0 (3.06–39.41)	0.631	0.72 (0.19–2.78)
*LIF*	<0.001	5.14 (1.96–13.47)	0.002	4.80 (1.77–13.0)
*MIEN1*	0.018	12.93 (1.55–107.9)	0.110	0.17 (0.02–1.49)
*PDCD1*	0.007	5.38 (1.58–18.27)	0.134	2.59 (0.75–8.98)
*RAD51B*	<0.001	7.91 (2.53–24.75)	0.001	6.81 (2.14–21.62)
*SLC2A3*	0.043	2.98 (1.03–8.62)	0.162	2.17 (0.73–6.44)

HR, hazard ratio; CR, complete response.

## Data Availability

The raw sequencing data supporting the findings of this study are restricted due to ethical considerations and are not publicly available. However, data can be requested from the corresponding author upon reasonable request. The analysis data necessary to reproduce the study are provided in Supplementary Tables.

## Supplementary Materials

The following supporting information can be downloaded at: https://www.mdpi.com/article/10.3390/ijms27208991/s1.
