# Supplementary material for: Prediction of Tumor Complete Response Using Genomic and Clinical Data in Solid Cancer Undergoing Definitive Radiation Therapy
Source: Int J Mol Sci. 2026 Oct 10;27(20):8991. doi: 10.3390/ijms27208991 (PMC13705160; doi:10.3390/ijms27208991)
Supplement: Supplementary file 1 [file ijms-27-08991-s001.zip › Supplementary_Figures.pdf]

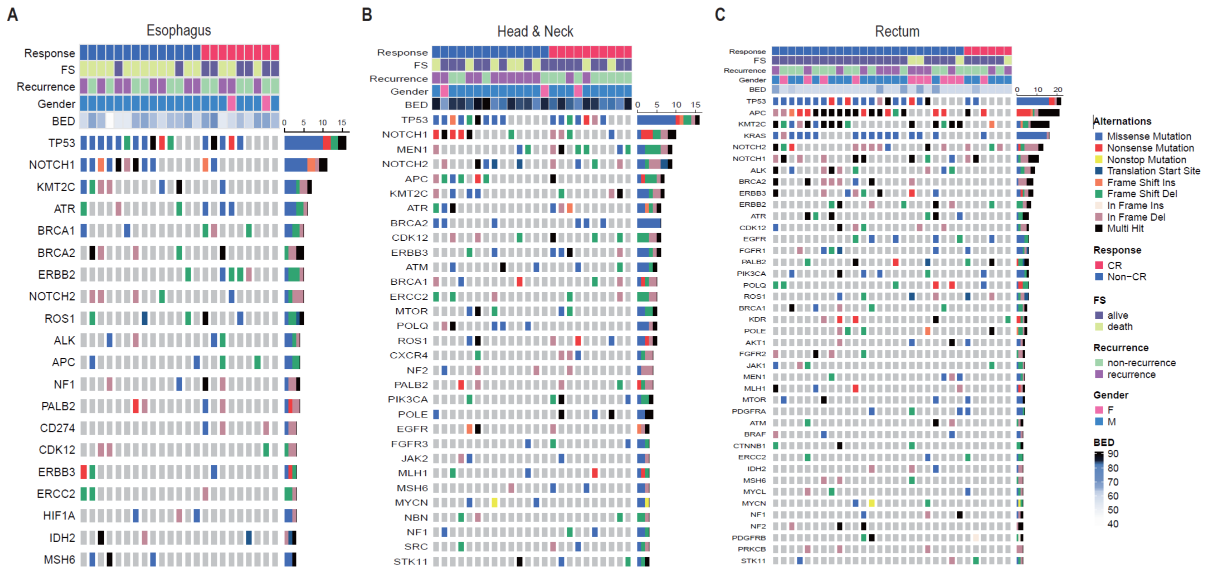

Figure S1. Cancer type stratified genomic alteration profiles. Oncoplots show selected genomic alterations in patients with (A) esophageal squamous cell carcinoma (ESCC; n = 23), (B) head and neck squamous cell carcinoma (HNSCC; n = 24), and (C) rectal adenocarcinoma (RAC; n = 30). Columns represent individual tumors, and rows represent genes. The upper annotation tracks indicate treatment response, final status, recurrence, gender, and biologically effective dose (BED). Colors indicate alteration types, and the bar plots on the right summarize the number of alterations for each gene. CR, complete response; FS, final status; BED, biologically effective dose.

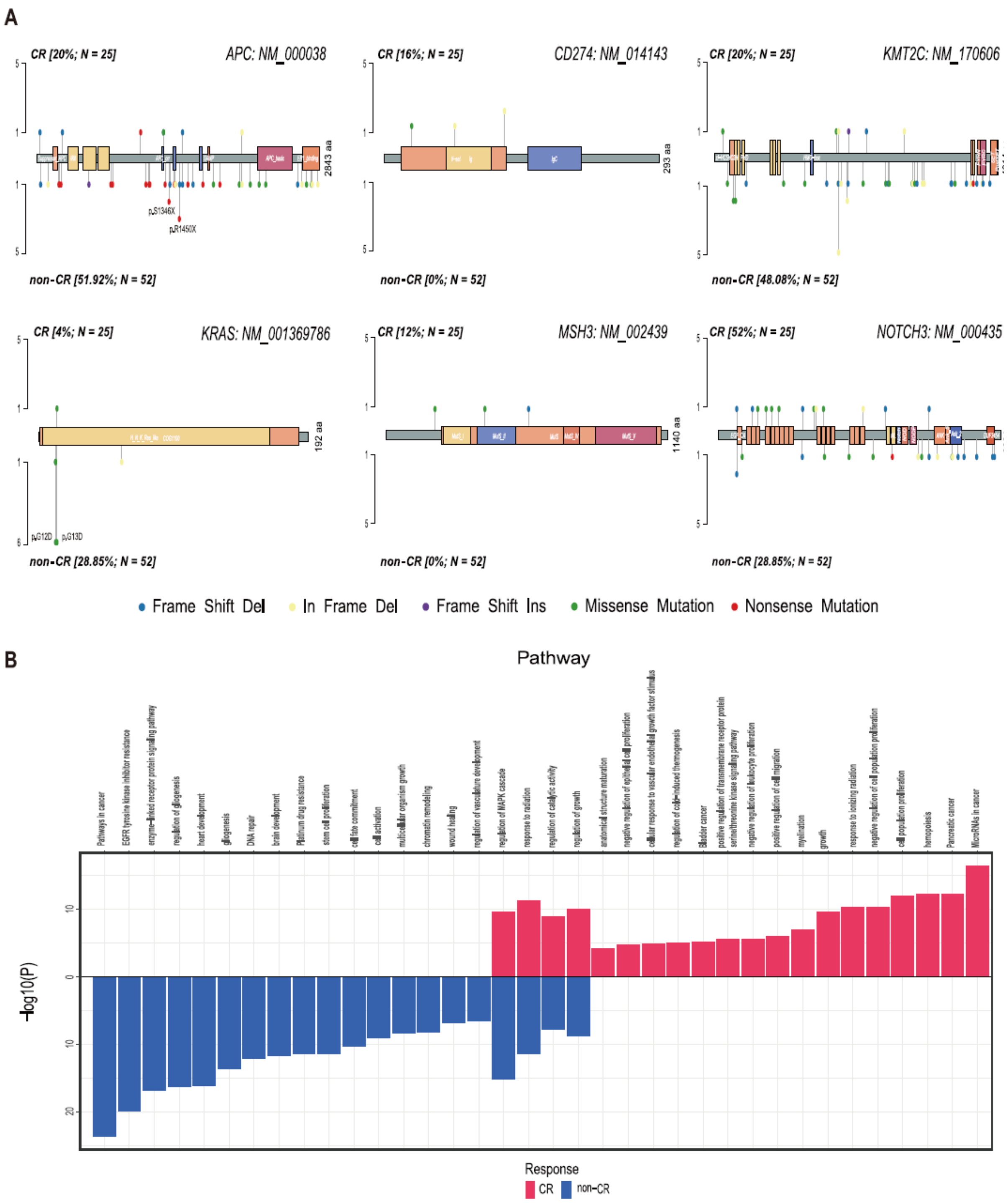

Figure S2. Genomic alterations and pathway enrichment according to radiation therapy response. (A) Lollipop plots showing the positions and types of alterations in *APC*, *CD274*, *KMT2C*, *KRAS*, *MSH3*, and *NOTCH3* in the CR and non-CR groups. Alterations identified in the CR group are shown above each gene structure, whereas those identified in the non-CR

group are shown below. The percentages indicate the proportions of patients with alterations in each gene within the corresponding response group, and colors indicate alteration types. (B) Pathway enrichment analysis using genes cataloged in the Catalogue of Somatic Mutations in Cancer (COSMIC) database and altered in ≥10% of patients within the CR or non-CR group. Bar length represents −log10(*P*). Pathways identified in the CR group are plotted above zero, whereas those identified in the non-CR group are plotted below zero for visualization. CR, complete response.

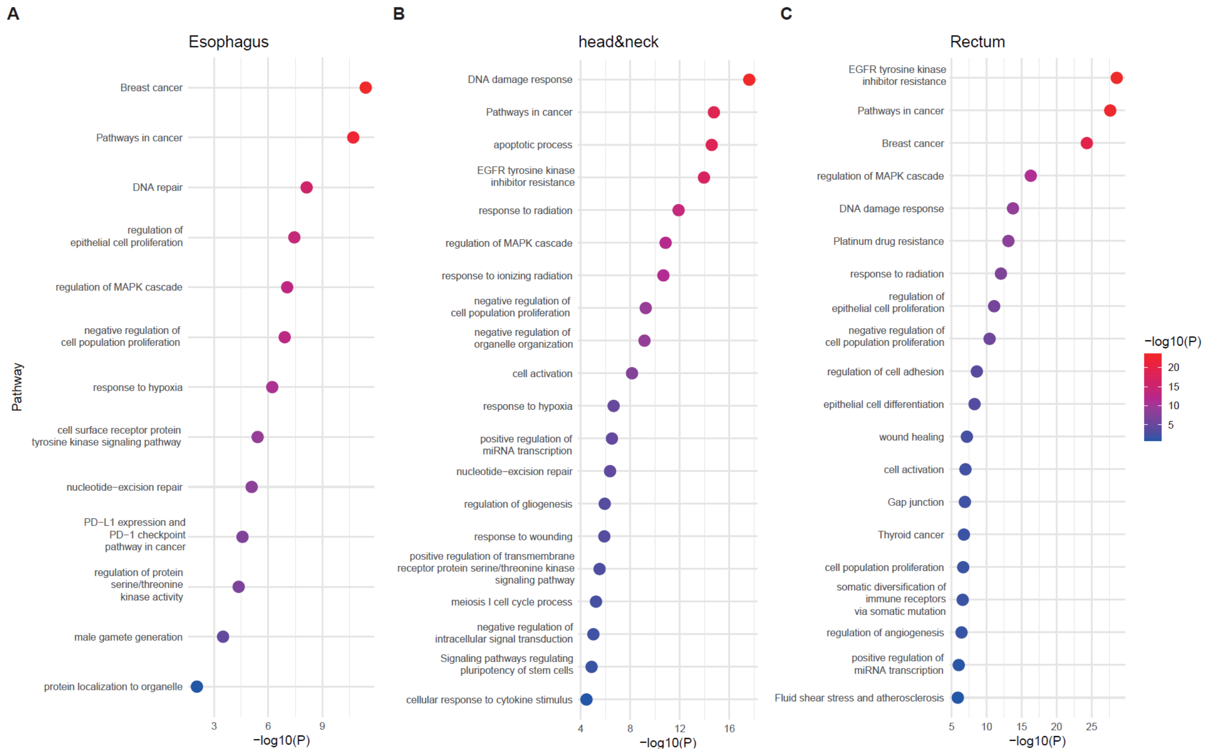

Figure S3. Cancer type stratified pathway enrichment analyses. Pathway enrichment analyses were performed separately for patients with (A) ESCC, (B) HNSCC, and (C) RAC using genes cataloged in the COSMIC database and altered in ≥10% of patients within each tumor type. The x-axis and point color represent −log10(P), with larger values indicating stronger enrichment. ESCC, esophageal squamous cell carcinoma; HNSCC, head and neck squamous cell carcinoma; RAC, rectal adenocarcinoma; COSMIC, Catalogue of Somatic Mutations in Cancer.

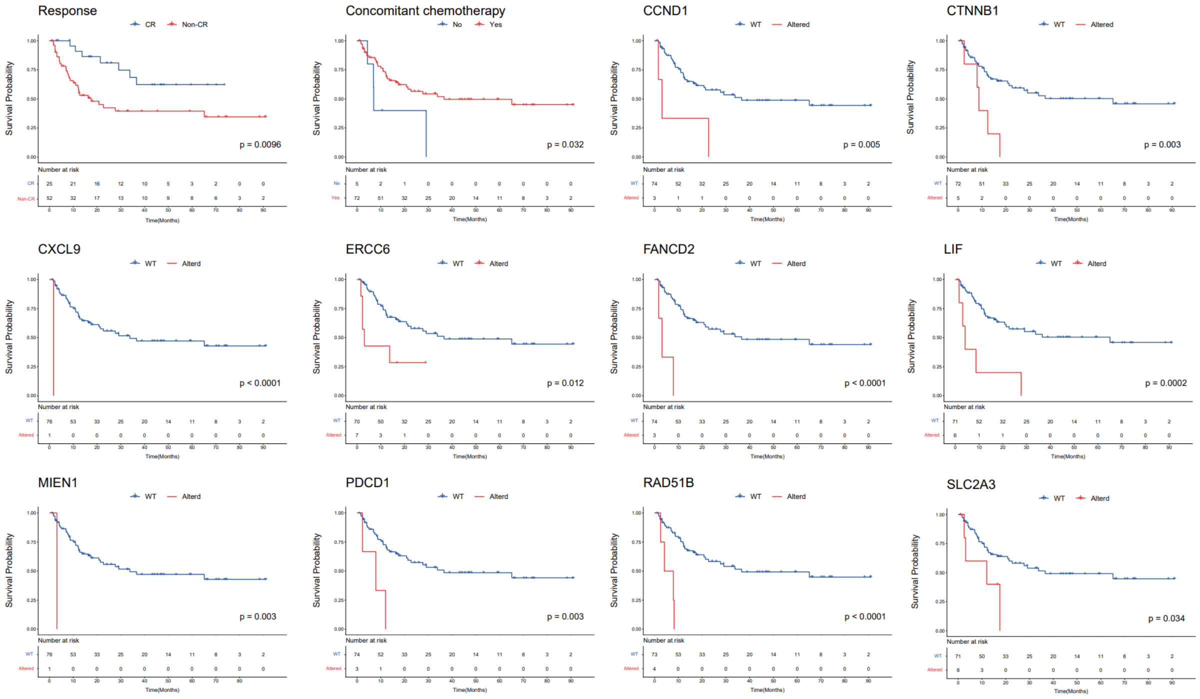

Figure S4. Kaplan–Meier curves for recurrence-free survival according to clinical and genomic factors. Recurrence-free survival was compared according to treatment response (CR vs. non-CR), concomitant chemotherapy (yes vs. no), and the alteration status of *CCND1*, *CTNNB1*, *CXCL9*, *ERCC6*, *FANCD2*, *LIF*, *MIEN1*, *PDCD1*, *RAD51B*, and *SLC2A3* (altered vs. wild-type). Numbers at risk are shown below each plot, and tick marks indicate censored observations. *P*-values were calculated using the log-rank test. CR, complete response; WT, wild-type.
